# Development and External Validation of an Explainable Machine-Learning Model for Predicting Postoperative Pulmonary Complications in Older Adults Undergoing Degenerative Spine Surgery

**DOI:** 10.3390/jcm15145375

**Published:** 2026-07-09

**Authors:** Ridan Lei, Jun Ou, Ye Chen, Manjun Luo, Xiaorui Ruan, Jianhui Wei, Ziye Li, Jiabi Qin

**Affiliations:** 1 Department of Epidemiology and Health Statistics, Xiangya School of Public Health, Central South University, Changsha 410013, China; 2 Department of Epidemiology and Health Statistics, School of Public Health, Kunming Medical University, Kunming 650500, China

**Keywords:** postoperative pulmonary complications, degenerative spine surgery, older adults, machine learning, prediction model, external validation, SHAP, decision curve analysis

## Abstract

**Background**: Postoperative pulmonary complications (PPCs) remain common in older adults undergoing degenerative spine surgery and are associated with adverse outcomes. We aimed to develop and externally validate an explainable machine-learning model using routinely available perioperative variables to estimate individual PPC risk. **Methods**: We conducted a two-center retrospective cohort study including consecutive patients aged ≥65 years undergoing degenerative cervical or lumbar spine surgery under general anesthesia. The development cohort included 1200 patients (Affiliated Nanhua Hospital, University of South China; 2024–2025), and the external validation cohort included 600 patients (First Affiliated Hospital, University of South China; 2025). PPCs within 7 postoperative days were defined using EPCO criteria. Twenty-five prespecified predictors were considered. Missing data were handled using multiple imputation, and continuous variables were standardized. Feature selection was performed using LASSO with 10-fold cross-validation and the 1-standard-error rule. We trained six machine-learning models (DT, RF, SVM, XGBoost, LightGBM, ANN) and logistic regression as a benchmark, with hyperparameters tuned by 5-fold cross-validation in the development cohort. Performance was assessed by AUROC, calibration (plots and Brier score), decision-curve analysis, and threshold-based metrics; SHAP was used for interpretability. A web-based calculator was implemented for clinical use. **Results**: PPCs occurred in 222/1200 (18.5%) patients in the development cohort and 123/600 (20.5%) in the external validation cohort. LASSO selected 11 predictors. In external validation, the random forest model achieved AUROC 0.786 (95% CI 0.740–0.829) and Brier score 0.137 (95% CI 0.119–0.154), with favorable decision-curve performance, and achieved an accuracy of 73.83%, sensitivity of 68.29%, specificity of 75.26%, PPV of 41.58%, NPV of 90.20%, and an F1 score of 51.69% at the prespecified threshold. **Conclusions**: We developed and externally validated multiple prediction models for PPCs in older adults undergoing degenerative spine surgery. The random forest model provided balanced performance and SHAP-based interpretability and was retained as an implementation-focused model for perioperative or immediate postoperative risk estimation. Further prospective validation, comparison with established clinical risk scores, and model updating in more diverse clinical settings are needed before routine clinical implementation.

## 1. Introduction

Postoperative pulmonary complications (PPCs) substantially worsen postoperative outcomes. Even mild PPCs have been associated with increased early postoperative mortality, higher overall morbidity, greater intensive care unit (ICU) utilization, and prolonged hospital length of stay [1,2]. Degenerative spine surgery in older adults often entails multilevel decompression and/or instrumented fusion, prolonged operative duration, and considerable perioperative physiological stress. These procedures may be accompanied by substantial blood loss and postoperative pain; opioid-based analgesia and delayed mobilization can further contribute to hypoventilation and impaired airway clearance, thereby increasing PPC risk [3,4]. Moreover, age-related reductions in pulmonary reserve and the high burden of cardiopulmonary comorbidities further heighten susceptibility to PPCs [5,6]. Despite their clinical importance, spine-specific estimates of PPC incidence in elderly degenerative spine surgery populations remain limited and vary across studies [7,8,9].

A systematic review has summarized multiple preventive strategies that may reduce PPC risk [10]. However, indiscriminate implementation of these measures can increase healthcare costs and may lead to inefficient use of limited resources. Timely identification of patients at high risk for PPCs is therefore essential to enable targeted prophylaxis, intensified monitoring, and efficient perioperative care.

Risk stratification for PPCs is a key priority in perioperative care. PPC risk reflects a complex interplay of baseline patient characteristics, comorbidities, and perioperative exposures [11,12,13]. However, existing prediction tools for PPCs have largely been developed in heterogeneous surgical populations or non-spine settings, and evidence specifically targeting older adults undergoing degenerative spine surgery remains scarce [14,15]. Consequently, a simple, clinically applicable, and externally validated model tailored to this high-risk population is currently needed.

In earlier work, predictive models have been predominantly developed using conventional statistical approaches such as logistic regression. While these methods are interpretable and widely used, they often rely on prespecified functional forms and may be less capable of capturing complex, nonlinear interactions among perioperative variables [16]. In addition, traditional modeling typically depends on manual feature specification and selection, which can be time-consuming and may introduce subjectivity, as it requires substantial domain expertise and predefined assumptions to construct and refine the model.

Accordingly, applying machine learning (ML) may help overcome these limitations and provide several practical advantages. First, ML models can capture nonlinear associations and high-order interactions in high-dimensional data, often with less reliance on prespecified functional forms or hand-crafted rules. Second, when appropriately trained and validated, ML approaches can achieve stable performance across different patient cohorts, supporting application to external clinical datasets. Third, modern ML pipelines are scalable to large datasets and can incorporate automated feature selection/representation learning, facilitating efficient model construction from heterogeneous perioperative and clinical variables [17]. Moreover, explainability techniques (e.g., SHAP) can improve model transparency and clinical interpretability.

Recent studies have applied ML to perioperative PPC prediction in general surgical and neurosurgical settings [18,19], with a limited number of studies extending to spine surgery populations, including spinal tumor surgery [20]. Nevertheless, evidence remains limited for ML-based PPC prediction specifically tailored to older adults undergoing degenerative spine surgery, a population characterized by reduced physiologic reserve, a high burden of comorbidities, and procedure-related stressors that may alter PPC risk profiles. Therefore, the present study aimed to identify perioperative predictors of PPCs and to develop and externally validate a two-hospital multicenter, explainable ML-based model for individualized PPC risk estimation in elderly patients undergoing degenerative spine procedures, with evaluation of discrimination, calibration, clinical utility (DCA), and model interpretability (SHAP).

## 2. Materials and Methods

### 2.1. Study Design and Ethics

This two-center retrospective cohort study for prediction-model development and external validation was approved by the Ethics Committee of the Affiliated Nanhua Hospital, University of South China (Approval No. 2024-ky-003) and was conducted in accordance with the Declaration of Helsinki. Given the retrospective design and the use of de-identified routinely collected data, the requirement for written informed consent was waived. The study is reported in accordance with TRIPOD recommendations for prediction model development and validation. The ethics approval was obtained retrospectively for the analysis of de-identified routinely collected clinical data. Data extraction and outcome adjudication were completed after the end of the study period, and the analytical dataset was locked before model development and external validation. Although the development and external validation cohorts partly overlapped in calendar time during 2025, they were derived from two independent hospitals with separate electronic medical record systems, and no patient could be included in both cohorts. According to available institutional records, no major formal changes in perioperative respiratory care protocols or postoperative monitoring pathways were identified during the study period.

### 2.2. Setting and Participants

**Figure 1:**
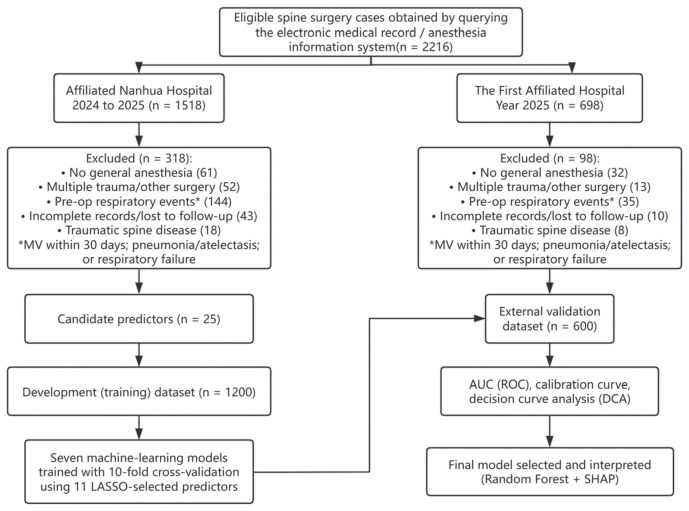
The overall flowchart of the study. * Preoperative respiratory events included invasive mechanical ventilation within 30 days before surgery, preoperative pneumonia/atelectasis, or a history of preoperative respiratory failure.

This two-hospital multicenter retrospective study included two hospitals. Consecutive eligible patients aged ≥65 years who underwent degenerative cervical or lumbar spine surgery under general anesthesia were enrolled. The development cohort was derived from the Affiliated Nanhua Hospital of the University of South China and included patients operated on between 1 January 2024 and 31 December 2025. An independent external validation cohort was assembled from the First Affiliated Hospital of the University of South China during 2025. The overall study workflow is shown in Figure 1.

Patients were identified from institutional electronic medical records and perioperative/anesthesia information systems. Inclusion criteria were: (1) age ≥ 65 years; (2) a primary diagnosis of degenerative spine-related disorders; and (3) cervical or lumbar spine surgery. Exclusion criteria were: (1) not receiving general anesthesia; (2) multiple trauma or concomitant operations involving other anatomical sites during the same admission; (3) severe preoperative respiratory compromise (invasive mechanical ventilation within 30 days before surgery, preoperative pneumonia or atelectasis, or a history of preoperative respiratory failure); (4) incomplete medical records or loss to follow-up within the 7-day outcome window; and (5) traumatic spinal conditions, such as acute spinal cord injury or spinal fractures.

### 2.3. Candidate Predictors and Data Sources

Perioperative data were retrieved from the electronic medical record and anesthesia/perioperative information systems at each participating hospital. Extracted information included baseline demographics, comorbidities, preoperative vital signs and laboratory tests (most recent results prior to surgery), surgical details, intraoperative management, and early postoperative documentation. Guided by previously reported PPC risk factors, clinical expertise, and routine data availability, we prespecified 25 candidate predictors potentially related to PPCs after degenerative spine surgery (Table S1). Candidate predictors covered: (1) demographics and baseline status (e.g., age, sex, BMI, ASA class); (2) comorbidities and preoperative conditions (e.g., COPD, heart failure, diabetes, CAD, recent URI, preoperative SpO_2_ < 96%, anemia, hypoalbuminemia, smoking status); (3) laboratory markers (e.g., WBC, CRP, Na, K); and (4) surgical/intraoperative factors (e.g., surgical region/type, segments ≥ 3, operation duration, estimated blood loss, crystalloid/colloid volumes).

### 2.4. Intended Timing of Prediction

Because several predictors, including operation duration, estimated blood loss, and intraoperative fluid administration, become available during or immediately after surgery, the primary model was intended for perioperative or immediate postoperative risk estimation rather than purely preoperative prediction. The intended use of the model is to support early postoperative monitoring, respiratory care planning, and allocation of preventive resources after intraoperative information becomes available.

### 2.5. Definitions for Comorbidities/Labs

For consistency across centers, comorbidities were identified based on documented physician diagnoses and/or active treatment recorded in the medical chart. Coronary artery disease was defined as a history of ischemic heart disease (e.g., prior myocardial infarction, angina, coronary revascularization) or a clinician-documented diagnosis. Definitions for anemia and hypoalbuminemia were prespecified a priori and are provided in the Supplement (Table S1).

### 2.6. Outcome Definition and Adjudication

Postoperative pulmonary complications (PPCs) were assessed within 7 postoperative days according to EPCO criteria [21]. Respiratory infection/pneumonia was considered present when antibiotics were administered for suspected respiratory infection and at least one of the following was documented: newly developed/worsened sputum production (often requiring nebulization therapy or sputum suction), new or progressive pulmonary infiltrates/opacities, body temperature > 38 °C, or leukocytosis (WBC > 12 × 10^9^/L). Respiratory failure was defined as clinically significant hypoxemia requiring escalation of oxygen therapy or advanced respiratory support, including any of the following: PaO_2_ < 90 mmHg or SpO_2_ < 95% while receiving 3 L/min oxygen via nasal cannula; SpO_2_ < 90% on room air necessitating supplemental oxygen; or escalation to advanced respiratory support such as noninvasive ventilation, reintubation, or tracheostomy. Suspected pleural effusion, atelectasis, pneumothorax, or aspiration pneumonitis required radiographic confirmation by chest radiography or CT. Detailed operational definitions for PPC components are provided in Supplementary Table S2.

Outcome determination was conducted through a systematic review of the ICU information system and the integrated electronic medical record. Two independent investigators, blinded to predictor variables, adjudicated PPC outcomes for each patient. Any discordance was resolved by discussion, and a third investigator was consulted to reach a final consensus when necessary.

### 2.7. Data Preprocessing

Data were screened for duplicates and implausible values; flagged outliers were verified against source records and corrected when appropriate. Missingness was assessed for each predictor. Predictors with >20% missingness would be excluded a priori; in this study, all 25 prespecified predictors met this criterion and were retained: Sex_male (male sex), BMI_cat (BMI category), ASA_cat (ASA class), COPD, SpO2_low (preoperative SpO_2_ < 96%), Anemia, Hypoalbumin (hypoalbuminemia), Surg_region_lumbar (lumbar region), Surg_type_major (major procedure type), Segments_ge3 (segments ≥ 3), URI_1m (URI within 1 month), Heart_failure, Preop_resp_train (preoperative respiratory training), Smoking_status, Diabetes, CAD, Age, WBC, CRP, Na, K, Op_duration_min, EBL_ml, Crystalloid_ml, and Colloid_ml.

### 2.8. Missing Data & Standardization

Missing values were handled using multiple imputation by chained equations (MICE) in R (mice package), generating five imputed datasets (m = 5) with a fixed random seed (seed = 123) [22]. To minimize information leakage, imputation in the external validation cohort was performed separately within that cohort using the same set of predictors and imputation settings and did not use outcome information. Continuous variables were standardized using Z-score normalization based on the development cohort and then applied to the external validation cohort to ensure consistent scaling and stable model training [23]. Models were trained separately in each of the five imputed datasets, and for each individual, predicted probabilities were averaged across the five imputations for performance evaluation. The missing-data mechanism was considered likely to be missing at random because missingness was mainly related to routine laboratory testing and documentation processes rather than to unobserved values themselves [22]. Missingness was summarized separately in the development and external validation cohorts, and no prespecified predictor exceeded the predefined 20% missingness threshold. The missingness proportions for all candidate predictors are reported in Supplementary Table S3.

### 2.9. Feature Selection & Class Imbalance

The development cohort comprised 1200 patients, and the external validation cohort included 600 patients. Given the moderately imbalanced outcome incidence (~18–21%), no data rebalancing techniques (e.g., SMOTE [24]) were applied to avoid altering the underlying event distribution; model performance was additionally summarized using threshold-based metrics.

### 2.10. LASSO CV Folds

**Figure 2:**
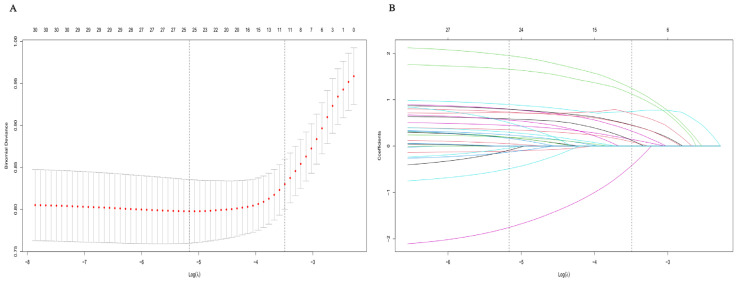
LASSO-based predictor selection for postoperative pulmonary complications. (**A**) Ten-fold cross-validation was used to select the optimal penalty parameter (λ) in the LASSO model. Points indicate the mean cross-validated error, and error bars represent ±1 standard error. The two vertical dashed lines denote λ_min (minimum error) and λ_1se (largest λ within 1 standard error of the minimum), respectively. (**B**) Coefficient trajectories of candidate predictors plotted against log(λ). As penalization increases, coefficients shrink toward zero, and variables with minimal contribution are eliminated; predictors with non-zero coefficients at the selected λ were retained for subsequent analyses. Different colors in panel B indicate different candidate predictors.

To derive a parsimonious predictor set, feature selection was performed within the development cohort using LASSO regression with 10-fold cross-validation (Figure 2). The final predictor set was selected using the 1-standard-error rule, and the resulting 11 predictors were carried forward unchanged for model training and external validation. Feature selection was performed within the imputed development data using the same LASSO procedure. To clarify the feature-selection process, the LASSO procedure was checked across the imputed datasets, and the selected predictor set remained clinically consistent with the final 11 predictors used for model training and external validation. These predictors were carried forward unchanged in all candidate models to ensure comparability across algorithms.

### 2.11. Model Training & Threshold

Using the selected predictors, we trained six machine-learning models—decision tree (DT) [25], random forest (RF) [26], support vector machine (SVM) [27], XGBoost [28], LightGBM [29], and artificial neural network (ANN) [30]—and additionally fitted logistic regression as a conventional benchmark model. Hyperparameters for each ML model were optimized via grid search with five-fold cross-validation (cv = 5) within the development cohort [31]. The optimal classification threshold was determined in the development cohort by maximizing Youden’s index on the ROC curve, and model-specific cut-offs were applied unchanged to the external validation cohort for threshold-dependent metrics. For the final selected random forest model, the cut-off was fixed at 0.226.

### 2.12. Performance Metrics

Model performance was evaluated across discrimination, calibration, and clinical utility. Discrimination was quantified using AUROC. Calibration was assessed using calibration plots and the Brier score. Clinical utility was evaluated using decision curve analysis (DCA) to estimate net benefit across a range of threshold probabilities. A confusion matrix was used to calculate accuracy, sensitivity, specificity, precision, and F1-score. The 95% confidence intervals (CIs) for AUROC and Brier score were estimated using bootstrap resampling (1000 replicates).

### 2.13. Model Interpretability (SHAP)

After selecting the final model, we used Shapley additive explanations (SHAP) to quantify feature contributions and enhance interpretability [32,33]. Global explanations were summarized using SHAP beeswarm and mean absolute SHAP bar plots. Local explanations were generated using SHAP force plots to illustrate how key features increased or decreased predicted PPC probability for individual patients. Building on the SHAP-based interpretation of the 11 selected predictors, we further implemented the final model as a Streamlit-based web calculator, enabling clinicians to obtain individualized PPC risk estimates conveniently in real time.

### 2.14. Statistical Analysis

Continuous variables are summarized as mean (standard deviation) or median (interquartile range), as appropriate, and categorical variables as counts (percentages). Baseline characteristics are presented descriptively for the development and external validation cohorts. Model performance metrics are reported with 95% confidence intervals where applicable. Analyses were conducted with SPSS (version 27.0; IBM Corp, Armonk, NY, USA), R (version 4.4.0), and Python (version 3.12.5).

## 3. Results

### 3.1. Cohort Assembly

A total of 2216 potentially eligible spine surgery cases were identified by querying the electronic medical record and anesthesia information systems. For model development, 1518 patients treated at the Affiliated Nanhua Hospital, University of South China from 2024 to 2025 were screened; 318 patients were excluded because they did not receive general anesthesia (n = 61), had multiple trauma/other surgery (n = 52), had preoperative respiratory events (mechanical ventilation within 30 days, pneumonia/atelectasis, or respiratory failure; n = 144), had incomplete records/loss to follow-up (n = 43), or had traumatic spine disease (n = 18). The remaining 1200 patients constituted the development cohort.

For external validation, 698 patients undergoing spine surgery at the First Affiliated Hospital, University of South China in 2025 were assessed; 98 patients were excluded for the same reasons (no general anesthesia, n = 32; multiple trauma/other surgery, n = 13; preoperative respiratory events, n = 35; incomplete records/loss to follow-up, n = 10; traumatic spine disease, n = 8), leaving 600 patients in the validation cohort.

### 3.2. Outcome Incidence and Baseline Characteristics

The primary endpoint was the occurrence of any postoperative pulmonary complication (PPC) within 7 postoperative days, defined according to the EPCO criteria. PPCs occurred in 222/1200 (18.5%) patients in the development cohort and 123/600 (20.5%) in the external validation cohort. Outcome adjudication was performed by two independent investigators blinded to predictor information, using standardized chart review procedures; disagreements were resolved by consensus (see Section 2). Baseline clinical characteristics are summarized in Table 1.

### 3.3. Missing Data Reporting

Missing values were observed in several predictors; however, no prespecified predictor exceeded 20% missingness, and all 25 candidate predictors were retained. The proportion of missing data for each predictor in both cohorts is summarized in Table S3 (range: 0.0–19.0%; among predictors with missing data, 3.8–19.0%). Missing values were addressed using MICE (m = 5, seed = 123) as described in the Methods.

### 3.4. Predictor Selection Using LASSO Regression for PPC After Spine Surgery

To obtain a parsimonious predictor set and reduce the risk of overfitting, we performed LASSO-penalized logistic regression on the 25 candidate variables in the development cohort (n = 1200). The tuning parameter λ was selected by 10-fold cross-validation (Figure 2A). Using the 1-standard-error rule, λ_1se = 0.03047 was chosen. At this penalty level, 11 predictors had non-zero coefficients and were retained for subsequent model development: age, serum sodium (Na), ASA class, COPD, heart failure, preoperative SpO_2_ < 96%, preoperative anemia, upper respiratory infection within 1 month before surgery, surgical segments ≥ 3, intraoperative estimated blood loss, and operation time (Figure 2B).

### 3.5. Multimodel Integrated Analysis for Classification

**Figure 3:**
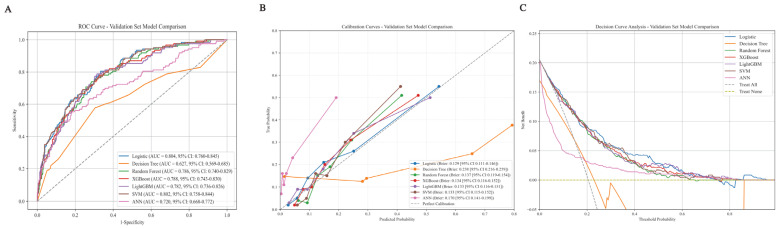
External validation performance of candidate models. (**A**) ROC curves for predicting postoperative pulmonary complications (PPCs) after spine surgery (n = 600). (**B**) Calibration curves comparing predicted and observed risks; the diagonal line indicates perfect calibration. (**C**) Decision curve analysis (DCA) showing net benefit across threshold probabilities (“treat-all” and “treat-none” as reference strategies).

Seven algorithms (logistic regression, decision tree, random forest, XGBoost, LightGBM, SVM, and ANN) were trained in the development cohort and evaluated in an independent external validation cohort. Discrimination, calibration, and clinical utility in the external validation cohort are summarized in Figure 3 (ROC, calibration plot, and DCA) and Table 2. In the external validation cohort, the random forest model showed good discrimination with an AUROC of 0.786 (95% CI 0.740–0.829) and achieved an accuracy of 73.83%, sensitivity of 68.29%, specificity of 75.26%, precision of 41.58%, and an F1 score of 51.69% at the classification threshold derived in the development cohort. Calibration was acceptable, with a Brier score of 0.137 (95% CI 0.119–0.154). Logistic regression and SVM yielded slightly higher AUROCs (0.804 and 0.802, respectively), whereas the decision tree showed substantially lower discrimination (AUROC 0.627) (Table 2). Because the external validation cohort was intended primarily for independent performance assessment, the comparison among algorithms should be interpreted as exploratory rather than as a formal procedure for selecting a universally superior model. In external validation, logistic regression and SVM achieved slightly higher AUROCs than random forest, whereas the random forest model showed the highest F1 score and acceptable calibration and decision-curve performance. Therefore, the random forest model was retained as an implementation-focused explainable model, while the comparative performance of all candidate models is reported transparently.

### 3.6. Interpretability and Application of the Model

**Figure 4:**
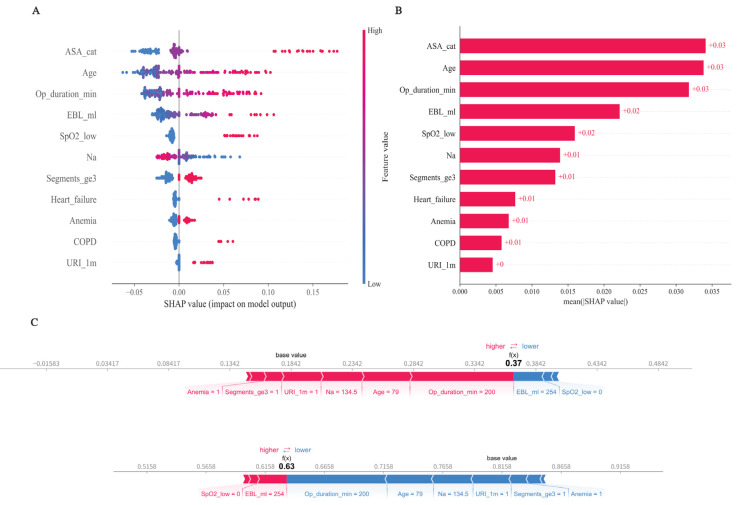
SHAP interpretation of the final model for postoperative pulmonary complications (PPCs). (**A**) SHAP summary (beeswarm) plot (red = high value, blue = low value). (**B**) Feature importance (mean |SHAP|). (**C**) Example force plots showing how features increase (red) or decrease (blue) the predicted PPC risk relative to the baseline. In SHAP plots, each SHAP value represents the contribution of a given feature to the model output for an individual patient. Positive SHAP values push the prediction toward a higher estimated PPC risk, whereas negative SHAP values push the prediction toward a lower estimated PPC risk. In the beeswarm plot, each point represents one patient, and the color indicates the feature value, with red representing higher values and blue representing lower values.

To improve the interpretability of the final model, SHAP (SHapley Additive exPlanations) was used to quantify the contribution of each predictor to the predicted risk of postoperative pulmonary complications (PPCs) after spine surgery. As shown in Figure 4B, global feature importance ranked by mean absolute SHAP values indicated that ASA class, age, and operation duration were the leading contributors, followed by estimated blood loss, preoperative SpO_2_ < 96%, serum sodium (Na), and surgical segments ≥ 3, with additional effects from heart failure, preoperative anemia, COPD, and upper respiratory infection within 1 month. The SHAP beeswarm plot (Figure 4A) further illustrates both the direction and magnitude of each variable’s impact: higher ASA class, older age, longer operation time, greater blood loss, presence of preoperative hypoxemia, more extensive surgery, and comorbid cardiopulmonary conditions generally shifted predictions toward a higher PPC risk, whereas higher sodium values tended to push predictions in the opposite direction. Finally, representative SHAP force plots (Figure 4C) provide patient-level explanations by showing how individual features collectively move the predicted probability above or below the model’s baseline output (red features increase the predicted risk, whereas blue features decrease it).

### 3.7. Web-Based Risk Calculator and Model Deployment

To enhance clinical usability and facilitate translation, we implemented the PPCs prediction model as an interactive web-based risk calculator using Streamlit. The application provides structured input fields for all model predictors (ASA class, age, operative duration, estimated blood loss, serum sodium, preoperative SpO_2_ < 96%, surgical segments ≥ 3, heart failure, preoperative anemia, COPD, and recent upper respiratory infection) and returns real-time individualized risk estimates after users submit patient-specific values by clicking the “Predict” button. The web tool is publicly accessible at https://ppcs-risk-predictor-febwf2nje3pnnm5bgl8zxa.streamlit.app/ (accessed on 7 June 2026), and the source code is available at https://github.com/173850711-max/ppcs-risk-predictor (accessed on 7 June 2026) to support transparency and reproducibility.

## 4. Discussion

In this two-hospital multicenter retrospective study, we developed and externally validated machine-learning models to predict PPCs in older adults undergoing degenerative spine surgery using routinely available perioperative variables. PPC incidence was approximately 18–21% across cohorts, highlighting the clinical importance of timely risk stratification. Among seven candidate algorithms, the random forest model demonstrated balanced performance in external validation across discrimination, calibration, and decision-curve analyses, and its predictions were further supported by SHAP-based interpretability. These findings suggest that ML-based risk estimation may help identify patients at higher risk who could benefit from targeted monitoring and preventive strategies [1,2,10].

SHAP analyses highlighted clinically plausible determinants of PPC risk. Higher ASA class and older age were dominant contributors, consistent with reduced physiologic reserve and increased comorbidity burden in older surgical patients [34,35,36,37]. Markers of surgical invasiveness and exposure—longer operative duration, greater blood loss, and more operated segments—also contributed substantially, aligning with prior evidence linking prolonged surgery and higher blood loss to PPC risk [38,39]. Several of these factors may be partly modifiable through perioperative planning, blood-conservation strategies, and early postoperative mobilization and respiratory care, which may help reduce pulmonary morbidity in high-risk individuals [40,41,42,43,44].

Preoperative physiologic status and comorbidity-related factors also played important roles, including reduced preoperative oxygen saturation, COPD, heart failure, anemia, and recent upper respiratory infection. Lower room-air SpO_2_ is an established predictor of PPCs and may reflect subclinical respiratory impairment [45,46]. ASA class and cardiopulmonary comorbidities have also been consistently associated with PPCs [36,46,47]. Because a proportion of this risk is potentially amenable to intervention, optimization of pulmonary disease, individualized decision-making for patients with a recent respiratory infection, and guideline-directed management of heart failure may be clinically relevant components of a risk-reduction strategy [45,48,49,50]. Similarly, identifying and treating anemia before surgery may improve physiologic reserve and reduce perioperative risk [51,52].

Serum sodium status contributed to risk stratification. Preoperative hyponatremia has been associated with increased postoperative adverse events, including pneumonia and other respiratory complications, and may serve as a readily available marker of underlying illness severity and reduced physiologic reserve [53,54,55,56,57]. Accordingly, perioperative assessment of etiology and individualized fluid and electrolyte management may be informative, particularly in older adults with multiple comorbidities.

In external validation, the random forest model achieved acceptable discrimination (AUROC 0.786) and satisfactory calibration (Brier score 0.137), with balanced threshold-based metrics and favorable decision-curve performance. Logistic regression and SVM showed slightly higher AUROCs; therefore, the comparison among candidate algorithms should be interpreted cautiously. In the present study, the random forest model was retained as an implementation-focused explainable model because of its balanced threshold-dependent performance, compatibility with SHAP-based interpretation, and potential usability in a web-based calculator, rather than because it was universally superior to all other models [58].

Our study extends prior work on PPC prediction that has largely relied on conventional models or risk scores developed in heterogeneous surgical populations [59,60,61,62,63,64]. By focusing on older adults undergoing degenerative spine surgery and providing independent external validation with explainability and an accessible web tool, our approach may better support individualized risk assessment in this high-risk subgroup. The ARISCAT score is a widely used clinical tool for estimating the risk of postoperative pulmonary complications in general surgical populations [11,12]. However, it was not specifically developed for older adults undergoing degenerative spine surgery and does not incorporate several spine-surgery-related intraoperative factors, such as operative duration, estimated blood loss, or surgical extent, which were important contributors in the present study. A direct head-to-head comparison with ARISCAT was not performed because not all ARISCAT variables were consistently available in the retrospective datasets. This lack of direct benchmarking against established clinical risk scores should be considered a limitation, and future prospective studies should compare the proposed model with ARISCAT and other established perioperative risk tools. Nonetheless, several limitations should be acknowledged. First, because this was a retrospective study, outcome ascertainment may have been influenced by clinical documentation, antibiotic initiation, oxygen-escalation practices, and the availability or timing of chest imaging. Although PPC outcomes were adjudicated by two independent investigators using predefined operational criteria, residual ascertainment bias cannot be excluded, and PPCs occurring after discharge or beyond the 7-day window may have been missed. Second, because operative duration and estimated blood loss were included as predictors, the model should be regarded as a perioperative or immediate postoperative risk-estimation tool rather than a purely preoperative prediction model. Third, although the validation cohort was independent, both hospitals were affiliated with the same university system and located in the same geographical region; therefore, further validation in more diverse clinical settings is needed. Finally, the comparison among candidate algorithms should be interpreted as exploratory. Logistic regression and SVM achieved slightly higher AUROCs than random forest, and the random forest model was retained mainly as an implementation-focused explainable model with balanced overall performance and SHAP-based interpretability, rather than as a universally superior model.

From an implementation perspective, because operative duration and estimated blood loss are included as predictors, the web-based tool may be more suitable for perioperative or early postoperative risk estimation once intraoperative information is available, rather than purely preoperative prediction. The predicted risk may help inform risk-stratified monitoring and preventive planning according to local resources and clinical priorities. Predicted risk estimates should complement, not replace, clinical judgment, and SHAP explanations should be interpreted cautiously, particularly when predictors are correlated.

## 5. Conclusions

In summary, we developed and externally validated multiple prediction models using multicenter perioperative data to estimate the risk of postoperative pulmonary complications in older adults undergoing degenerative spine surgery. The random forest model was retained as an implementation-focused explainable model with balanced overall performance and SHAP-based interpretability. The model may support individualized PPC risk estimation and risk-stratified postoperative monitoring, but further prospective validation, comparison with established clinical risk scores, and model updating in more diverse clinical settings are needed before routine clinical implementation.

## Figures and Tables

**Table 1:** Patient characteristics of the development and external validation cohorts.

Characteristics	Development Cohort (n = 1200)	External Validation Cohort (n = 600)
**Age (years) ^a^**	74.4 ± 5.8	74.0 ± 5.8
**WBC (×10^9^/L) ^a^**	6.5 ± 1.5	6.5 ± 1.4
**CRP (mg/L) ^a^**	7.3 ± 14.5	6.5 ± 8.8
**Na (mmol/L) ^a^**	138.6 ± 2.9	138.4 ± 2.8
**K (mmol/L) ^a^**	4.1 ± 0.3	4.1 ± 0.4
**Estimated blood loss (mL) ^a^**	406.5 ± 347.5	397.9 ± 350.3
**Operation time (min) ^a^**	124.2 ± 42.7	125.7 ± 44.1
**Crystalloid (mL) ^a^**	1623.2 ± 490.6	1610.9 ± 500.3
**Colloid (mL) ^a^**	191.3 ± 307.5	199.7 ± 304.8
**BMI (kg/m^2^) ^b^**		
<18.5	80 (6.7%)	40 (6.7%)
18.5–23.9	594 (49.5%)	287 (47.8%)
24–27.9	382 (31.8%)	200 (33.3%)
≥28	144 (12.0%)	73 (12.2%)
**ASA ^b^**		
1–2	477 (39.8%)	243 (40.5%)
3	606 (50.5%)	287 (47.8%)
4–5	117 (9.8%)	70 (11.7%)
**Sex ^b^**		
Male	625 (52.1%)	295 (49.2%)
Female	575 (47.9%)	305 (50.8%)
**Smoking status ^b^**		
Never	791 (65.9%)	395 (65.8%)
Quit ≥4 weeks	183 (15.3%)	93 (15.5%)
Quit 2–4 weeks	55 (4.6%)	37 (6.2%)
Quit <2 weeks	41 (3.4%)	23 (3.8%)
Current smoker	130 (10.8%)	52 (8.7%)
**Preop SpO_2_ ^b^**		
<96%	141 (11.8%)	70 (11.7%)
≥96%	1059 (88.3%)	530 (88.3%)
**Surgical region ^b^**		
Cervical	314 (26.2%)	162 (27.0%)
Lumbar	886 (73.8%)	438 (73.0%)
**Surgery type ^b^**		
Decompression only	566 (47.2%)	265 (44.2%)
Fusion/instrumentation/revision	634 (52.8%)	335 (55.8%)
**Surgical segments ^b^**		
1–2	616 (51.3%)	299 (49.8%)
≥3	584 (48.7%)	301 (50.2%)
**COPD ^b^**	85 (7.1%)	31 (5.2%)
**Diabetes ^b^**	392 (32.7%)	211 (35.2%)
**Coronary artery disease ^b^**	328 (27.3%)	160 (26.7%)
**Heart failure ^b^**	74 (6.2%)	40 (6.7%)
**Preop anemia ^b^**	428 (35.7%)	201 (33.5%)
**Preop hypoalbuminemia ^b^**	198 (16.5%)	112 (18.7%)
**URI within 1 month ^b^**	83 (6.9%)	56 (9.3%)

^a^ Mean ± standard deviation. ^b^ Percentage (%). BMI, body mass index; WBC, white blood cell count; CRP, C-reactive protein; Na, sodium; K, potassium; SpO_2_, peripheral oxygen saturation; COPD, chronic obstructive pulmonary disease; ASA, American Society of Anesthesiologists physical status classification; URI, upper respiratory infection. Categorical variables are presented as n (%). For binary variables, counts and percentages refer to the ‘Yes’ category unless otherwise specified. Bold entries indicate variable group headings.

**Table 2:** Performance of candidate models in the external validation cohort.

	XGB	SVM	LGBM	LR	RF	DT	ANN
Accuracy	72.83%	66.17%	72.83%	70.67%	73.83%	67.17%	81.33%
Sensitivity	69.92%	81.30%	68.29%	76.42%	68.29%	57.72%	16.26%
Precision	40.57%	35.71%	40.38%	39.00%	41.58%	32.87%	68.97%
Specificity	73.58%	62.26%	74.00%	69.18%	75.26%	69.60%	98.11%
F1 score	51.34%	49.63%	50.76%	51.65%	51.69%	41.89%	26.32%
NPV	90.46%	92.81%	90.05%	91.92%	90.20%	86.46%	81.96%
AUC (95% CI)	0.788 (0.743–0.830)	0.802 (0.758–0.844)	0.782 (0.736–0.826)	0.804 (0.760–0.845)	0.786 (0.740–0.829)	0.627 (0.569–0.685)	0.720 (0.668–0.772)

Values are reported for the external validation cohort. Accuracy, sensitivity, specificity, precision (positive predictive value, PPV), negative predictive value (NPV), and F1 score are presented as percentages (%). AUC is presented with the 95% confidence interval (CI). Abbreviations: AUC, area under the receiver operating characteristic curve; CI, confidence interval; XGB, eXtreme Gradient Boosting; SVM, support vector machine; LGBM, Light Gradient Boosting Machine; LR, logistic regression; RF, random forest; DT, decision tree; ANN, artificial neural network. Threshold-dependent metrics were computed using model-specific cut-offs derived in the development cohort and fixed in external validation; for the final random forest model, cut-off = 0.226.

## Data Availability

The datasets analyzed during the current study are not publicly available due to institutional restrictions but are available from the corresponding author on reasonable request, subject to approval by the participating institutions.

## Supplementary Materials

The following supporting information can be downloaded at https://www.mdpi.com/article/10.3390/jcm15145375/s1. Table S1: Candidate predictors and coding definitions; Table S2: Operational definitions of postoperative pulmonary complications based on EPCO criteria and related perioperative outcome definitions [21]; Table S3: Missingness of candidate predictors in the development and external validation cohorts.
